# Detection of GPS Spoofing Attacks in UAVs Based on Adversarial Machine Learning Model

**DOI:** 10.3390/s24186156

**Published:** 2024-09-23

**Authors:** Lamia Alhoraibi, Daniyal Alghazzawi, Reemah Alhebshi

**Affiliations:** Faculty of Computing and Information Technology, King Abdulaziz University, Jeddah 21589, Saudi Arabia; dghazzawi@kau.edu.sa (D.A.); ralhebshi@kau.edu.sa (R.A.)

**Keywords:** spoofing attack, physical layer security, adversarial machine learning, generative adversarial networks

## Abstract

Advancements in wireless communication and automation have revolutionized mobility systems, notably through autonomous vehicles and unmanned aerial vehicles (UAVs). UAV spatial coordinates, determined via Global Positioning System (GPS) signals, are susceptible to cyberattacks due to unencrypted and unauthenticated transmissions with GPS spoofing being a significant threat. To mitigate these vulnerabilities, intrusion detection systems (IDSs) for UAVs have been developed and enhanced using machine learning (ML) algorithms. However, Adversarial Machine Learning (AML) has introduced new risks by exploiting ML models. This study presents a UAV-IDS employing AML methodology to enhance the detection and classification of GPS spoofing attacks. The key contribution is the development of an AML detection model that significantly improves UAV system robustness and security. Our findings indicate that the model achieves a detection accuracy of 98%, demonstrating its effectiveness in managing large-scale datasets and complex tasks. This study emphasizes the importance of physical layer security for enhancing IDSs in UAVs by introducing a novel detection model centered on an adversarial training defense method and advanced deep learning techniques.

## 1. Introduction

The progressions in technology have permeated nearly all aspects of human life. With the evolution of wireless communication and automation technologies, there is a significant transformation in mobility systems. A notable trend toward increased automation and connectivity among vehicles is exemplified by the emergence of autonomous vehicles and UAVs. The UAV industry has garnered considerable investment since its initiation in the current decade. According to a report from Precedence Research [1], the UAV market achieved an estimated valuation of approximately USD 31 billion in 2023 and is anticipated to reach a valuation of approximately USD 100 billion by 2030. This expansion can be ascribed to widespread UAV adoption for military applications [2,3] as well as civilian applications, such as traffic monitoring [4,5], disaster monitoring and management [6,7], construction management [8], delivery services [9], and search and rescue operations [10].

UAV spatial coordinates, including location and altitude, are determined through the reception of satellite signals by the Global Navigation Satellite System (GNSS). The GNSS provides satellite-based positioning, navigation, and timing (PNT) services, which are more commonly referred to as the GPS. The GPS infrastructure consists of a constellation of satellites originating from the US Government’s Department of Defense in 1970 for military applications [11]. Analogously, several nations have independently developed their own satellite systems to provide PNT services. The original implementation of GPS catered predominantly to military objectives; however, by 1980, this technology was extended for civilian utilization on a worldwide basis. The transmitters deployed on GPS satellites generate distinct signals designed for military and civilian applications. Military signals, characterized by encryption, possess enhanced security compared to their civilian counterparts, which remain unencrypted. Many civilian GPS systems employ non-secure communication protocols, including the unencrypted and unauthenticated transmission of GPS signals. Consequently, the communication channels of these systems, established with other network entities over wireless mediums, are vulnerable to cyberattacks.

GPS signals are susceptible to both unintentional and intentional vulnerabilities [12,13]. Unintentional vulnerabilities manifest when a GPS signal becomes inaccessible, particularly when navigating tunnels; thick clouds in the sky may attenuate the signal strength, causing service interruptions. Tall buildings contribute to multipath propagation, leading to radio frequency interference and the subsequent degradation of signal strength. Natural events can also result in data integrity loss by adding noise to the signal. However, intentional vulnerabilities can be categorized into three primary types of attacks [14], as follows:

- Data interception attacks refer to unauthorized access to confidential information and are primarily attributable to the absence of robust encryption and authentication methods. Passive eavesdropping threatens privacy and network confidentiality, wherein adversaries clandestinely monitor communication traffic between entities.
- Data manipulation attacks undermine the integrity of information by altering existing data, fabricating new information, or employing a combination of both techniques. These attacks include deletion, replaying pre-recorded packets, injecting new packets, and direct modification.
- Denial of service (DoS) attacks obstruct communication systems within a network from transmitting and receiving data by inundating targets with many deceptive signals.

A notable example of a data manipulation attack is GPS spoofing, which is a systematic attack aimed at disseminating inaccurate information to GPS receivers. GPS spoofing is achieved through transmitting signals that emulate authentic GPS signals or by capturing legitimate GPS signals from a distinct location and time and retransmitting them [15]. Thus, the ensuing spoofing attack leads GPS receivers to receive false position and time information.

Security is a critical challenge affecting all emerging wireless technologies, including UAVs. IDSs have emerged as fundamental instruments for safeguarding wireless network security. Accordingly, unmanned aerial vehicle-based intrusion detection systems (UAV-IDSs) are developed through automated analysis to identify abnormal behaviors or malicious activities within a network [16]. UAV-IDSs examine the signals, behavior, control instructions, command traffic, power consumption, and operations of UAV components [17]. An IDS typically comprises three primary components: data collection, vectorization, and a classifier [18]. The classifier, which constitutes the core of the IDS, assesses whether the transformed vector meets the intrusion criteria. Recently, IDS classifiers have increasingly leveraged ML algorithms to enhance classification performance.

The open-source nature of ML techniques and the unencrypted characteristics of GPS signals have drawn the attention of adversaries aiming to exploit UAV system vulnerabilities. These adversaries may utilize ML techniques to manipulate GPS signal information, presenting significant challenges for conventional detection systems in identifying and mitigating such deceptive activities. Despite the success of ML for IDSs, the advent of Adversarial Machine Learning (AML) has recently emerged as a significant threat to the effectiveness of such systems. An adversary can exploit vulnerabilities in the ML algorithm or the trained ML model to compromise network defense.

The increase in AML attacks has motivated the scientific community to conduct more research to create and implement new defense strategies to counteract these attacks. An adversarial learning method [19] is an attempt to improve the reliability of the ML model by training it with adversarial samples. The adversarial training defense method enhances the model’s robustness by utilizing a training dataset containing benign and adversarial samples. In contrast, employing AML methodologies within detection systems as a defensive strategy seeks to enhance identification resilience by incorporating training datasets that include benign and adversarial samples.

The UAV networks use several IDS approaches for protection, including rule-based IDSs [20], signature-based IDSs [21], and anomaly-based IDSs [22,23]. Rule-based and signature-based IDSs predominantly identify known threats, while anomaly-based IDSs detect abnormal behaviors. Regardless of the current capabilities of IDS approaches, given the pressing need for optimization and robustness in UAV-IDSs, this study aims to fill this gap by exploring an innovative approach for evaluating and enhancing the capabilities of a ML-based IDS in identifying new and emerging threats. In addition, in this study, we highlight the effectiveness of AML methodology in improving the strength of ML and deep learning (DL) models when confronted with attacks involving adversarial signals, contributing to the development of more robust and secure UAV systems in the real world.

The main contributions of this study are as follows:

- This study develops an AML detection model encompassing both offensive and defensive dimensions to strengthen the efficacy of GPS spoofing attack detection within UAV-IDSs.
- Furthermore, this study uses an adversarial training method to generate robust adversarial samples to evaluate the performance of a DL-based IDS.
- This study implements experiments to provide a thorough understanding of the proposed model’s effectiveness and the performance of DL-based classification models, namely convolutional neural networks (CNNs) and long short-term memory (LSTM), in the context of GPS spoofing attacks.
- This study systematically utilizes various evaluation methods and metrics to assess the proposed model’s performance.

The remainder of this paper is organized as follows: Section 2 discusses relevant work on the ML-based detection of wireless signal spoofing and GPS spoofing. Section 3 describes the system model. Then, in Section 4, an AML detection model is proposed. Section 5 details the implementation of the proposed model. Section 6 presents the experimental results. Section 7 describes the paper’s limitations and future directions. Finally, Section 8 presents the paper’s conclusions.

## 2. Related Works

Considering the increasing vulnerability to wireless signal spoofing, numerous methods have been proposed for identifying and mitigating such attempts. This section examines select studies that have proposed ML-based approaches for detecting wireless signals and GPS spoofing attacks.

### 2.1. ML-Based Detection Wireless Signal Spoofing

Erpek et al. [24] used an AML approach to design jamming attacks and mitigation strategies. They developed a defense mechanism where the transmitter deliberately takes incorrect actions in specific time slots to mislead jammers. They used a generative adversarial network (GAN) to speed up learning before the attack. The study showed that introducing a small percentage of deliberate errors in selected time slots can significantly increase jammer errors, protecting the transmitter’s performance. Shi et al. [25] proposed a GAN-based spoofing attack for wireless security, where a deep neural network (DNN) generates synthetic signals mimicking legitimate ones, making them hard to distinguish. They used a pre-trained deep learning classifier to identify signals without spoofing and considered two baseline attacks: random signal transmission and replaying real signals. Their evaluation showed the high success rate of the GAN-based attack across different network topologies.

Conversely, in their research, Ma et al. [26] used a Recurrent Neural Network (RNN) to develop an intrusion detection method with Channel State Information (CSI) for secure and reliable performance. They employed LSTM as a classifier to differentiate legitimate users from malicious intruders and enhance detection efficiency. Orthogonal frequency division multiplexing was used for signal modulation and demodulation, and additive white Gaussian noise was included in the communication simulation. The authentication algorithm, evaluated with three users at varying signal-to-noise ratios (SNRs), achieved 100% accuracy in distinguishing users across different SNR levels.

### 2.2. ML-Based Detection GPS Spoofing

Different studies have proposed ML-based approaches for GPS spoofing detection. Manesh et al. [27] proposed a neural network-based method to detect GPS spoofing messages. They analyzed five key features: satellite number, carrier phase, pseudo-range, Doppler shift, and SNR, to optimize accuracy and minimize false alarms. The study also evaluated the performance of different neural network configurations and numbers of hidden neurons. Aissou et al. [28] proposed four supervised tree-based machine learning models—Random Forest (RF), Gradient Boost, XGBoost, and LightGBM—to detect GPS spoofing attacks and select suitable unmanned aerial system (UAS) models. All models detected GPS spoofing attacks in under 22 milliseconds, but XGBoost outperformed the others in accuracy, detection time, and memory usage. XGBoost was three times faster and used half the memory, making it ideal for UASs due to its size, weight, and power constraints. Shafique et al. [29] developed a model to detect counterfeit GPS signals triggered by UAVs upon receiving GPS signals. Their study addresses mitigating GPS spoofing attacks on UAVs by employing multiple ML algorithms to find the best classification algorithm. They used GPS signal characteristics as features and improved their method’s precision by creating various ML models through K-fold analyses with different K values. The resulting K-learning models were used for voting, using both soft and hard voting mechanisms to classify test data. The proposed model’s effectiveness was validated through experiments, demonstrating its robustness and practical potential.

Li et al. [30] introduced an approach to spoofing signal detection under the single-peak condition by combining GAN with traditional signal processing techniques. They focus on detecting residual signals during the acquisition phase and study cases with minimal differences between spoofing and authentic signals. Simulations show that their method improves detection efficacy, especially when the spoofing signal’s pseudo-code phase and carrier Doppler frequency are similar to authentic signals. Khoei et al. [31] proposed two dynamic-based selection methods—Metric-Optimized Dynamic (MOD) and Weighted MOD—for detecting GPS spoofing attacks on UAVs. They applied ten popular supervised ML classifiers: Support Vector Machine (SVM), Naive Bayes (NB), Decision Tree (DT), K-Nearest Neighbors (KNN), linear discriminative analysis, Random Forest (RF), Artificial Neural Network (ANN), logistic regression, Elastic Net, and AdaBoost. Training and testing were conducted using a dataset with 13 GPS signal features, and both methods achieved high accuracy.

Sun et al. [32] developed a new model for detecting GPS signal spoofing using a combination of principal component analysis, CNN, and LSTM neural networks. They collected real GPS signal data with a composite-wing UAV and addressed the small dataset issue by applying the SVM-SMOTE model to enhance the data. The model achieved a high accuracy rate, demonstrating its effectiveness in spoofing detection. Borhani-Darian et al. [33] investigated using a DNN for detecting GNSS spoofing attacks. They applied a cross-ambiguity function based on I/Q samples from GNSS receivers to identify spoofing. An image-splitting technique created an efficient data-driven classifier with parallel processing. Their Gaussian mixture model approach determined the optimal number of spoofing signals. The results show that their deep learning method outperforms existing techniques, especially in moderate-to-high SNR conditions.

Table 1 presents detailed information on the reviewed detection wireless signal spoofing-based machine learning approaches, including the algorithms used, features, solutions, and other remarks.

Situated within the expanding field of AML methodology, this study contributes a novel perspective on signal spoofing detection tailored to UAV security. Given the pressing need for optimization and robustness in this area, this study aims to fill this gap by exploring innovative approaches for detecting and mitigating wireless physical layer threats in UAVs, which is guided by the unique characteristics of the signals and AML methodology. Although the use of generative models in signal spoofing detection is still limited, this study takes a pioneering step by employing the GAN model to augment datasets and optimize the proposed detection model. The model is not static but dynamic, continuously refined, and reassessed using additional training and test datasets to ensure its effectiveness. Driven by GAN’s ability to estimate complex probability distributions, this iterative approach instills confidence in the model’s robustness. The insights derived from this study enhance the effectiveness of AML methodology in strengthening IDS robustness in cybersecurity.

## 3. System Model

The absence of encryption in civilian GPS signals serves as a magnet for adversaries seeking to exploit vulnerabilities in UAVs. One prevalent cyber threat that has emerged due to this vulnerability is GPS spoofing, wherein adversaries manipulate GPS signals to deceive and compromise UAV navigation systems. The adversary propagates inaccurate signals within this context, reproducing legitimate satellite signals. The UAV processes a discrete-time GPS signal obtained through sampling from N satellites [33] as follows:

(1) $$y\left[n\right]=\sum_{i=1}^{N}x_{i}\left[n;\theta_{i}\right]+\eta \left[n\right]$$

(2) $$x_{i}\left[n;\theta_{i}\right]=\alpha_{i}b_{i}\left(nT_{s}-\tau_{i}\right)c_{i}\left(nT_{s}-\tau_{i}\right)\;e^{j2\pi f_{d}nT_{s}+j\phi_{i}}$$

Various parameters can describe the GPS signal from the *i*-th satellite. These include the amplitude $\alpha_{i}$, the navigation message $b_{i}$, the satellite’s spreading code $c_{i}$, the time delay $\tau_{i}$, the Doppler frequency $f_{d}$, the carrier phase $\phi_{i}$ introduced by the transmission channel, and the random noise η[n] present at the receiver.

In contrast, the GPS signal received during a spoofing attack can be described as follows:

(3) $$y\left[n\right]=\sum_{i=1}^{N}x_{i}\left[n;\theta_{i}\right]+\sum_{j=1}^{N_{s}}x_{j}\left[n;\theta_{s,j}\right]+\eta \left[n\right]$$

In this context, $N_{s}$ represents the total number of spoofed signals. For the receiver to be misled, each spoofed signal must replicate the satellite’s spreading code and transmit a legitimate navigation message. The spoofed amplitude, code phases, and carrier phases are collected and denoted by $\theta_{s,j}$ for the *j*-th spoofing source.

Thus, developing a GPS spoofing detection model can be framed as a hypothesis-testing scenario in which two competing hypotheses are evaluated.

- The null hypothesis (H0) suggests that the legitimate signal and noise exist without a spoofed signal.
  (4) $$H_{0}:y\left[n\right]=\sum_{i=1}^{N}x_{i}\left[n;\theta_{i}\right]+\eta \left[n\right]$$
- The alternative hypothesis (H1) posits the presence of both legitimate and spoofed signals alongside noise.
  (5) $$H_{1}:y\left[n\right]=\sum_{i=1}^{N}x_{i}\left[n;\theta_{i}\right]+\sum_{j=1}^{N_{s}}x_{j}\left[n;\theta_{s,j}\right]+\eta \left[n\right]$$

In this study, we introduced two conceptual architectures. The first architecture focuses on UAV ground control stations (GCSs) equipped with IDS to identify GPS spoofing. We also assumed a scenario in which an adversary launches an evasion attack by disseminating GPS-spoofed signals to deceive the signal detection classifier. In the second architecture, we enhanced the IDS capability by integrating it with the proposed AML detection model.

### 3.1. Offensive Scenario

**Figure 1:**
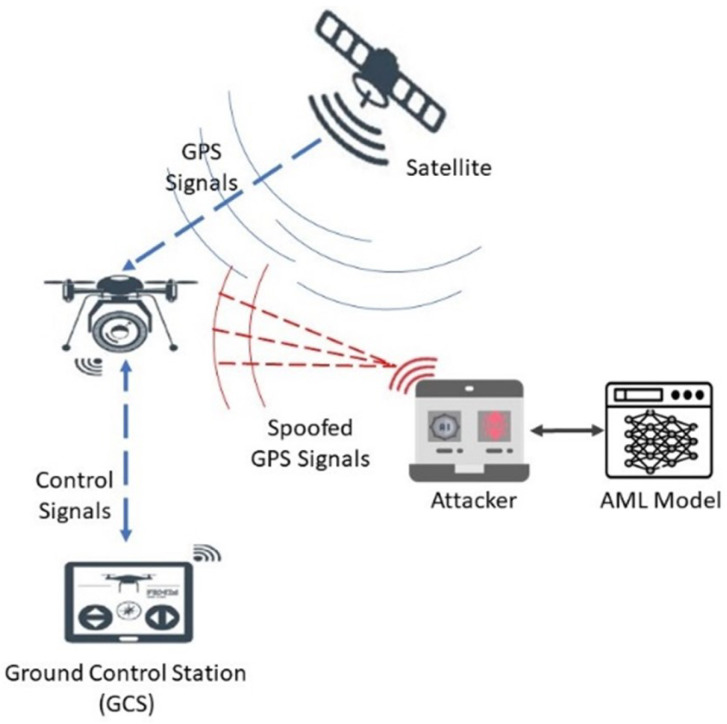
Overview of the offensive architecture.

Figure 1 illustrates an advanced form of a GPS spoofing attack. In an offensive scenario, the adversary aims to observe the activities within wireless communications and indirectly seeks to influence UAV behavior. The adversary strategy involves the manipulation of GPS signals with the overarching objective of compromising the integrity of the UAV’s detection system. The proposed offensive model involves an adversary who can disseminate manipulated GPS signals using the ML model and an external antenna to deceive nearby UAVs. The adversary fabricates fake GPS signals by analyzing the distinctive characteristics of legitimate GPS satellite signals and broadcasting them within the targeted area. This type of spoofing is highly intricate, posing a challenge in identifying legitimate signals.

### 3.2. Defensive Scenario

**Figure 2:**
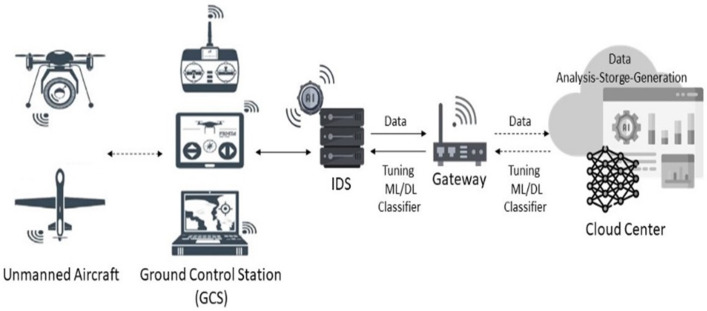
Overview of the defensive architecture.

Figure 2 illustrates the proposed architecture for the UAV-IDS in the current study. The system involves a GCS as a real-time decision center for managing multiple UAVs. The GCS is advantageous because of its capacity to offer significant computing resources and operate IDSs based on ML and DL algorithms. Upon applying the proposed model, the UAV is prepared to undertake appropriate actions in response to a declaration affirming the legitimacy of the received GPS signal from a satellite. Conversely, if GPS signal spoofing is detected, the UAV refrains from taking further action and awaits subsequent incoming signals. This process continues iteratively until the UAV successfully acquires a legitimate GPS signal, at which point it proceeds to execute the appropriate action.

## 4. Proposed AML Detection Model Architecture

This section introduces a detection model centered on AML training methodology based on the principles of DL techniques. To maintain the robustness of the UAV-IDS-based DL model against evasion attacks, we recognize the potential for adversaries to utilize generative models to simulate legitimate signals, inducing misclassification by the signal classifier. Consequently, we introduce an AML detection model to enhance the efficacy of UAV-IDS by providing an AML training methodology encompassing both defensive and offensive frameworks.

In contrast to traditional training methodologies, adversarial training introduces a novel approach involving self-penetration testing. This method aims to discern vulnerabilities inherent within the IDS classifier by capitalizing on adversarial samples. Such an approach facilitates the refinement process through iterative tuning to enhance the robustness and efficiency of the classifier.

### 4.1. Defensive Framework

**Figure 3:**
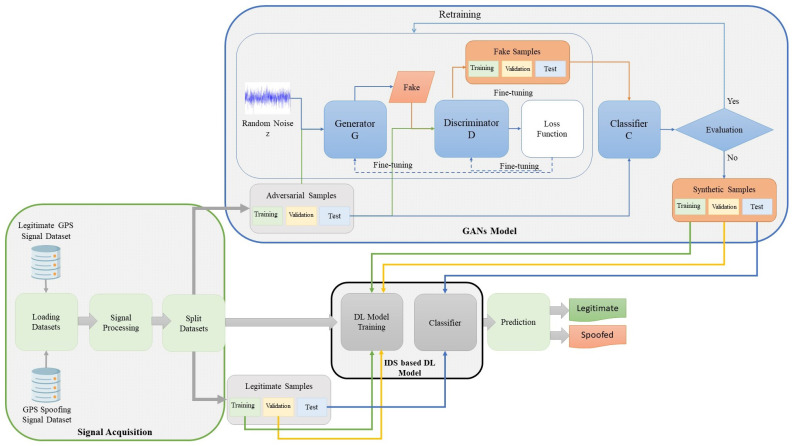
Proposed detection model.

In the defensive scenario, we propose the detection model explained in Figure 3, which seeks to strengthen the robustness of IDS through integration with the GAN model. A structured three-phase methodology implements the presented model. The initial phase begins with signal acquisition and dataset loading, which is followed by feature extraction processing. We systematically partitioned the dataset into training, testing, and validation sets and trained two baseline IDSs founded on DL models, specifically CNN and LSTM. We then carefully tested the GAN model’s creation of various adversarial samples against the established baseline models. Finally, in the third phase, we evaluated the robustness of the IDS classifier.

#### 4.1.1. Phase 1: Baseline Training

Initially, two distinct datasets are utilized: one containing legitimate signals and the other containing spoofing signals. Subsequently, the data are partitioned into subsets designated for training, testing, and validation, which are used as inputs for DL models. During this phase, the training set facilitates the foundational training of DL models within the IDS. Simultaneously, the validation set evaluates the model’s performance on previously unseen data. The final step in this training phase is to test the IDS classifiers using dedicated test sets. Under these conditions, the IDS classifier is configured for binary classification to accurately differentiate between ‘Legitimate’ and ‘Spoofed’ signals.

#### 4.1.2. Phase 2: Generate an Adversarial Sample

Upon completing the DL model baseline training, a generative model is implemented in this phase to create synthesized signals that mimic spoofing signals. A GAN model was employed to overcome the generative complexity, leveraging GAN’s ability to assimilate cumulative effects from signals, learn their distribution during the training phase, and subsequently generate fake signals based on the acquired knowledge. In [34], we explained the GANs model configuration we followed in this framework.

GAN architecture generally comprises two fundamental networks: a generator (*G*) and discriminator (*D*). The generator imitates the distribution of real data (*x*) and generates fake data *G*(*z*), where *z* is the noise input to the generator. The purpose of the discriminator is to identify the differences between *G*(*z*) and *x*. The GAN model can be considered a min–max game in which the generator aims to minimize the generator loss function by generating indistinguishable data from real data. The second aim is to maximize the discriminator’s loss function. The formula for this learning process is as follows:

(6) $$min_{G}\;max_{D}\;f\left(D,G\right)=E_{x∼P_{data}\left(x\right)}\left[\log\left(D\left(x\right)\right)\right]+E_{z∼P_{z}\left(z\right)}[\log\left(1-D\left(G\left(z\right)\right)\right]$$

where *E_x_* is the expected value over all real data, *D*(*x*) is the discriminator’s estimate of the probability of real data, *E_z_* is the expected value over all *G*(*z*) values, and *D*(*G*(*z*)) is the discriminator’s estimate of the probability that *G*(*z*) is real. The two loss functions are derived by comparing the two probability distributions produced by the generator, *P_z_*, and the other from the real dataset, Pdata.

In the flow of the GAN model shown in Figure 3, the classifier (*C*) evaluates the degree of similarity between the fake adversarial samples generated by the GAN model and the real adversarial samples.

#### 4.1.3. Phase 3: Classification Evaluation

In the final phase of the proposed detection model, the effectiveness of the IDS classifier is systematically evaluated using a dataset containing adversarial samples. The integrated DL models within the IDS undergo iterative retraining with an augmented dataset, theoretically enhancing the model’s robustness. Periodic retraining is essential in adversarial wireless environments, as it is required in many IDSs due to the non-stationary characteristics of the wireless signal distribution over time. This necessity arises from the gradual shift in the signal distribution. Retraining is a powerful defense mechanism against adversaries, impeding their ability to understand and adapt to the detector by consistently updating the model.

### 4.2. Offensive Framework

**Figure 4:**
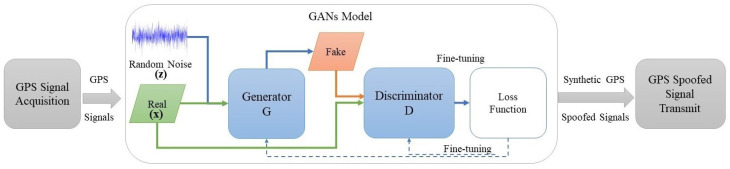
Proposed offensive model.

The process flow representation in Figure 4 shows the proposed offensive model. Initially, the adversary aims to capture legitimate signals by discerning the distinctive patterns inherent in these signals. This discernment facilitates the generation of false signals designed to replicate the identified patterns. The adversary attempts to produce a signal statistically similar to that received. However, generating such a signal proves complex without comprehensive knowledge. Accordingly, the basic architecture of the GAN model is employed to generate fake signals to overcome this complexity. The adversary initiates the training process by refining a *G* model, enhancing the synthesis of fake signals to make them statistically similar to legitimate signals. This optimization is pursued iteratively, increasing the classification error in the *D* model. The iterative process between *G* and *D* persists until a point of convergence is reached. Once the generator reaches convergence, the adversary can send fake signals that are similar to legitimate signals, making them easier to use for adversarial purposes.

## 5. Implementation

This section discusses the comprehensive implementation details of the proposed AML detection model.

### 5.1. GPS Spoofing Dataset Description

A dataset documenting GPS spoofing attacks is introduced in [35]. Real-time experiments and simulations were executed to collect a dataset containing legitimate and spoofed signals across various dates and locations. This dataset includes information from legitimate GPS signals obtained through an eight-channel GPS receiver atop a vehicle moving from 0 to 60 mph, simulating UAV flight. The dataset encompasses data from three stationary positions atop various buildings at varying altitudes to replicate UAV hovering operations. The associated dataset is structured to ensure balance across different types of GPS spoofing attack signals characterized by varying levels of complexity: simple, intermediate, and sophisticated.

- In a simple spoofing attack, spoofed signals are deliberately unsynchronized from legitimate signals. Consequently, a notable Doppler shift exceeds the standard range of ±20 Hz, leading to substantial variance in the pseudo-range measurement. In such instances, the adversary broadcasts the deceptive signal at a high power level, causing a heightened C/N° value.
- During an intermediate spoofing attack, the perpetrator possesses information about the UAV’s position, leading to code phase alignment between legitimate and spoofed signals. In this type of attack, careful management ensures that the Doppler shift and pseudo-range values remain within acceptable limits, preventing them from surpassing standard ranges.
- In a sophisticated attack, the adversary employs multiple synchronized antennas to mimic the GPS constellation. This strategy enables the adversary to simultaneously spoof various channels, attaining comprehensive control over the system.

This dataset comprises 510,530 samples, with 397,825 representing legitimate signals, while the remaining samples are evenly distributed among the three types of GPS spoofing attack signals. Within the dataset, there are 13 distinct features derived from various receiver stages from acquisition and tracking to the observable block. A comprehensive list and concise description of the received GPS signals’ features are presented in Table 2.

### 5.2. Hardware and Software Details

We trained the proposed model on a supercomputer equipped with Tesla K20 (NVIDIA, Santa Clara, CA, USA) graphical processing units, around 12,000 (Intel, Santa Clara, CA, USA) central processing unit cores, and a total RAM capacity of 96 GB across the system. We implemented the DL models using Python 3.10.9. We used packages such as Keras, TensorFlow, Sklearn, Matplotlib, NumPy, and SciPy to develop the Python scripts. We used MobaXterm v23.2 to execute Python scripts on the supercomputer.

### 5.3. DL Model Structures

This subsection provides the optimized parameter configuration for the GAN and DL models of the proposed model.

The generator’s structure is detailed in Table 3. Generating fake data begins by providing the generator with a random vector for shape (1,6), which represents a point in the latent space. The next layer in the structure is a dense layer containing six neurons with a linear activation function. This is followed by a reshape layer. A transpose convolutional layer is then applied to double the input size. The final layer is a convolutional layer with a single neuron. To determine the optimal activation function for the generator model, we used the linear activation function specifically within the dense layer. The output generated is a signal vector with a shape of (13).

Table 4 details the structure of the discriminator. Initially, the input vector has the dimensions of (1,13). The structure includes two convolutional layers, comprising 32 and 16 neurons. LeakyReLU serves as the activation function throughout these layers. The output from the convolutional layers is then transformed into a one-dimensional vector via a flattening layer. To mitigate overfitting, a dropout layer is incorporated. The final layer is a dense layer with a single neuron, classifying the data as real or fake.

Table 5 and Table 6 clarify the specifications and configurations of the DL architectures proposed for employment within the IDS classifier. The architecture encompasses two distinct models, namely CNN and LSTM, which are pivotal in facilitating the classification process.

Table 5 details the structure of the CNN classifier. Initially, the input vectors are loaded and reshaped into a fixed size of (1,1,13) for both the training and test datasets. The structure generally consists of three convolutional layers containing 64, 32, and 16 neurons, which is followed by a flattening layer. The CNN architecture’s final layer is a dense layer that uses sigmoid activation to perform signal classification. Table 6 details the structure of the LSTM classifier. The input vectors are first fed to all neurons in the LSTM model for classification. The first, second, and third layers contain 64, 32, and 16 neurons, respectively. LeakyReLU activation functions are used in all layers. In our case, we implemented three LSTM layers and connected each layer to subsequent layers that expected sequences as input, after which we set the return_sequences parameter to true. A flattening layer follows this, and the final layer is dense and uses sigmoid activation.

## 6. Experimental Results and Analysis

### 6.1. Experimental Setup

To evaluate AML detection model performance, we conducted a comprehensive analysis comprising four experiments:

- In the initial experiment, we assessed the performance of the IDS classifier based on CNN and LSTM architectures using a dataset containing 129,980 samples. This dataset comprised 66,256 legitimate GPS signals and 63,724 samples representative of three types of GPS spoofing attack signals.
- In the subsequent experiment, we selected the most optimal architecture based on the initial experiment results to assess the individual performance of each type of GPS signal spoofing attack. The first evaluation used a dataset with 83,079 samples of 46,621 legitimate signals and 36,458 simple spoofing attack signals. The second evaluation used a dataset with 88,901 samples of 44,668 legitimate signals and 44,233 intermediate spoofing attack signals. The last evaluation used a dataset with 77,114 samples, of which 45,101 were legitimate signals and 32,013 were sophisticated spoofing attack signals.
- The third experiment focused on enhancing the adaptability of the IDS classifier within the proposed AML detection model. We achieved this by utilizing the GAN model to generate 10,976 synthetic samples simulating a simple GPS spoofing attack, 8790 synthetic samples representing an intermediate GPS spoofing attack, and 9176 synthetic samples corresponding to a sophisticated GPS spoofing attack.
- The final experiment was crucial to ensure the robustness of the IDS classifier against evasion attacks. To achieve this, we generated 2000 synthetic samples from legitimate GPS signals.

### 6.2. Model Training Configuration Details

The training process of the AML detection model was conducted using the hyperparameters outlined in Table 7.

### 6.3. Evaluation Methods

**Figure 5:**
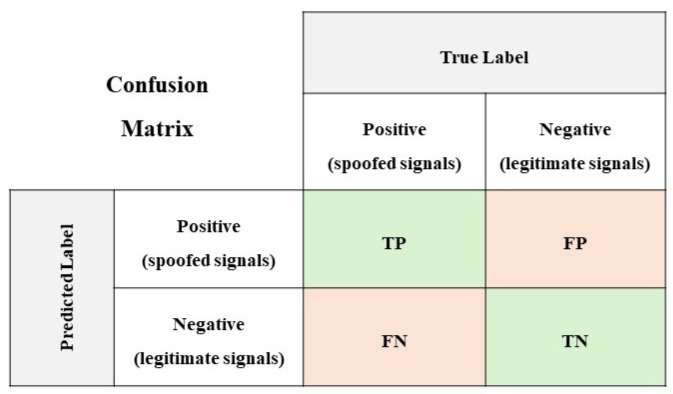
Binary confusion matrix.

To evaluate the performance of the DL models, it is beneficial to compute key metrics such as accuracy, precision, and recall, as these metrics provide valuable insights into the models’ effectiveness. The utilization of a confusion matrixis instrumental in this evaluation process. The confusion matrix serves as a metric for evaluating classification models, facilitating the determination of true positive and true negative values by collecting samples for actual and predicted labels. Figure 5 illustrates the binary confusion matrix for the reference.

The proposed models’ performance was assessed using several evaluation metrics. These metrics are derived from the confusion matrix properties, as follows: true positive (*TP*), false positive (*FP*), false negative (*FN*), and true negative (*TN*). The main metrics of this evaluation are as follows:

(7) $$Accuracy=\frac{\left(TP+TN\right)}{\left(TP+FP+FN+TN\right)}$$

(8) $$Precision=\frac{TP}{\left(TP+FP\right)}$$

(9) $$Recall=\frac{TP}{\left(TP+FN\right)}$$

(10) $$F1\;score=2\times \frac{Precision\times Recall}{Precision+Recall}$$

To assess the effectiveness of the GAN model, we followed a qualitative evaluation process. The qualitative assessment relied on human visual assessment to examine the generated samples.

**Figure 6:**
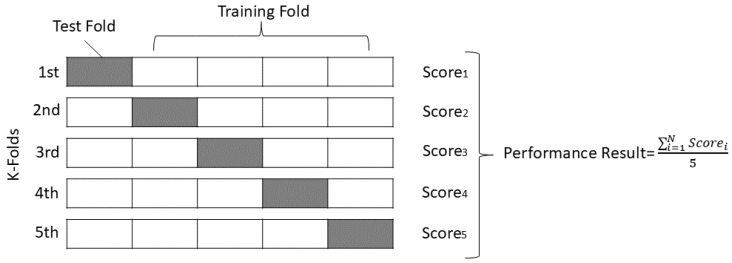
K-fold cross-validation analysis with five iterations chosen as K-fold.

To evaluate the performance of the DL models, we conducted K-fold cross-validation. The primary objective of utilizing the K-fold cross-validation technique is to ensure that every sample in the dataset undergoes testing. The number of iterations required to assess model performance via K-fold cross-validation is contingent on the K value. In our investigation of the AML detection model, we executed five iterations to evaluate the effectiveness of the proposed model. Figure 6 illustrates the validation processes for the five iterations. Following the K-fold validation of the dataset, we trained our proposed model to predict the nature of the signal, distinguishing between legitimate and spoofed signals.

### 6.4. Experiment 1

The initial experiment evaluated the effectiveness of the IDS classifier based on CNN and LSTM architectures. This experiment used a dataset containing legitimate GPS signals and various GPS signal spoofing attacks. The quantitative results, encompassing the scores of the diverse metrics, are shown in Table 8.

The primary aim of this experiment was to ascertain the optimal DL models for classifying GPS signals. Performance results must be analyzed to assess the effectiveness of DL models. In this context, the average loss for the CNN model was 0.14, whereas for the LSTM model, it was 0.52. The loss metric encapsulates the disparity between the predicted and actual values, serving as a critical indicator of model accuracy. Consequently, optimizing DL models requires minimizing this loss function to enhance the fidelity of predictive outcomes. In addition, the results showed that the CNN model attained the highest accuracy (93%), while the LSTM model achieved a comparatively lower accuracy (71%). The notable difference in accuracy between the CNN and LSTM models underscores the superior performance of the former. Furthermore, the analysis outcomes in Table 8 indicate the CNN model’s exceptional performance.

### 6.5. Experiment 2

Based on the results of the initial experiment, we selected the CNN model for the second experiment. This experiment assessed the CNN model’s performance on each type of GPS signal spoofing attack and included a comprehensive analysis of the results.

The findings presented in Table 9 indicate the strong performance of the CNN model in classifying distinct types of GPS signal spoofing attacks. Notably, the CNN model demonstrated a satisfactory average accuracy of 89% in distinguishing the simple GPS signal spoofing attack and a comparable average accuracy of 88% in classifying the intermediate type. However, the model showed slightly decreased performance in detecting sophisticated spoofing attacks with an accuracy of 83%. High precision indicates a low incidence of falsely classifying legitimate signals as spoofed signals. The model demonstrated a high precision of 95% in classifying intermediate and sophisticated types.

Conversely, the high recall value indicates a low incidence of misclassifying spoofed signals as legitimate signals. The model performed well when classifying the simple GPS spoofing attack with an average recall of 85%. In contrast, the model showed insignificant recall results, particularly in determining sophisticated types, where the average recall result achieved 64%, reflecting a reduction of approximately 20% compared to other attack types. The evaluation of the model’s performance shows an F1 score of 88% for both the simple and intermediate types; however, a notable decrease in model performance is observed when classifying sophisticated types, where the F1 score decreases to 75%.

### 6.6. Experiment 3

The main objective of the third experimental investigation was to assess the effectiveness of implementing the proposed AML detection model to enhance the IDS classifier capabilities. Table 10 shows the observed improvements in all metrics scores compared with the results presented in Table 9.

**Figure 7:**
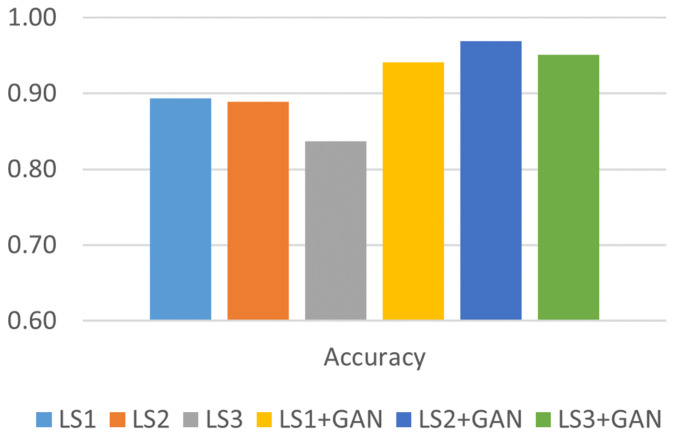
Accuracy analysis.

The comparative analysis depicted in Figure 7 shows notable enhancements in the model’s accuracy following the integration of synthetic GPS spoofing datasets for retraining the IDS classifier. Significantly, the average accuracy outcomes before implementing the AML detection model for all types of GPS signal spoofing attacks were below the 90% threshold.

After implementing the proposed AML model, the average accuracy substantially increased particularly in detecting sophisticated types of attacks. The accuracy in detecting simple GPS spoofing attacks increased from 89% to 94%. Similarly, the accuracy in identifying intermediate attacks improved from 89% to 97%. For sophisticated attacks, the accuracy increased from 84% to 95% after integrating the AML model.

**Figure 8:**
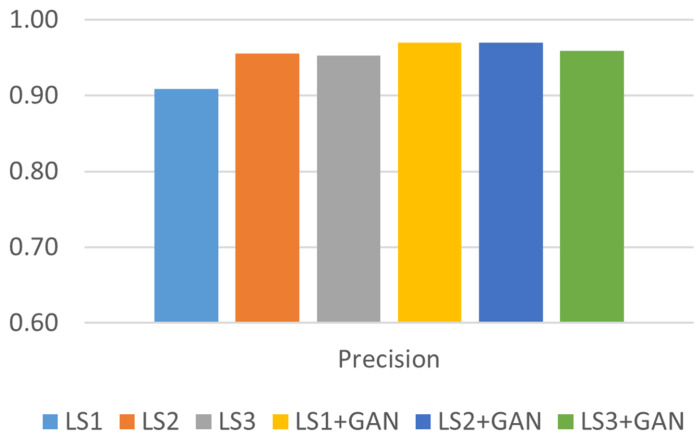
Precision analysis.

Precision is particularly significant in detection systems, where the ramifications of false positive predictions can lead to severe outcomes. Figure 8 shows that the proposed model produced advanced precision results before and after applying the AML detection model. The model showed precise predictions following the integration of the AML detection model, achieving an average precision of 97% for simple and intermediate types of GPS signal spoofing attacks and a comparable average precision of 96% for sophisticated types.

**Figure 9:**
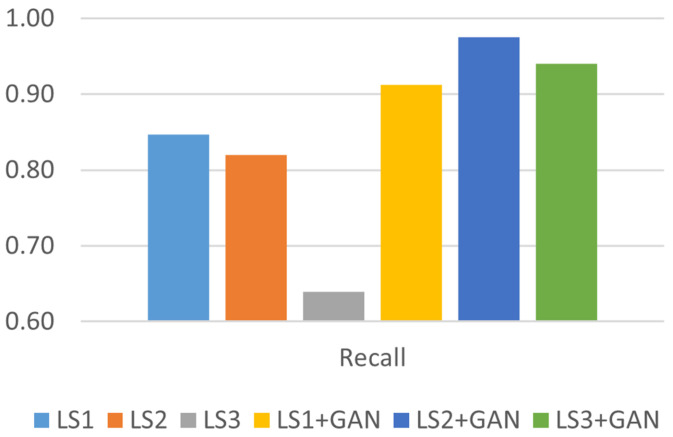
Recall analysis.

Figure 9 shows the notable improvement in recall after applying the AML detection model. A higher recall value indicates an improvement in model performance, achieving an average recall of 97% in detecting the intermediate type of GPS signal spoofing attacks and an average recall of 91% for simple types. There was a significant change in recall when the model was employed to detect sophisticated attacks, wherein the average accuracy registered an increase of approximately 30%.

**Figure 10:**
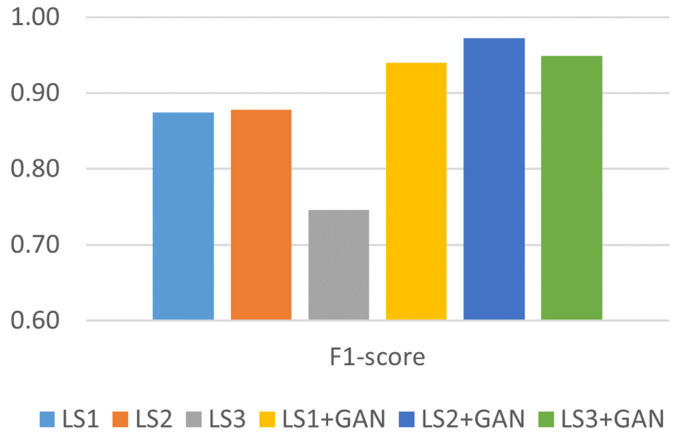
F1 score analysis.

Figure 10 provides a comprehensive depiction of the overall performance of the classification models. The F1 score increased when the proposed AML detection model was applied. The F1 score increased by 12%, on average, after integrating the proposed AML detection model.

### 6.7. Experiment 4

Experiment 4 assessed the effectiveness of incorporating an offensive model within the proposed AML detection mode to determine whether an offensive model could strengthen the proposed detection model’s robustness against evasion attacks. The assessment measured the detection model’s effectiveness and facilitated its refinement through iterative tuning processes. We hypothesized that the adversary would utilize a GAN model in this experimental study to create complex GPS spoofing signals. To test this, a dataset of 2000 samples was generated, and the effectiveness of the detection model was evaluated both before and after applying the AML detection model.

Table 11 demonstrates the robustness of the AML detection model in mitigating evasion attacks. Before applying the proposed detection model, many spoofed signals (748 instances) were misclassified as legitimate. This led to a classification accuracy result of 63% with a corresponding model loss result of 2.8. However, a marked decrease in misclassified signals was observed after implementing the proposed detection model. The model misclassified only 32 spoofed signals as legitimate, resulting in a classification accuracy of 98%. This significant improvement shows that the AML detection model provides strong protection against evasion attacks, making the classification process more accurate and trustworthy.

### 6.8. Model Assessment

**Figure 11:**
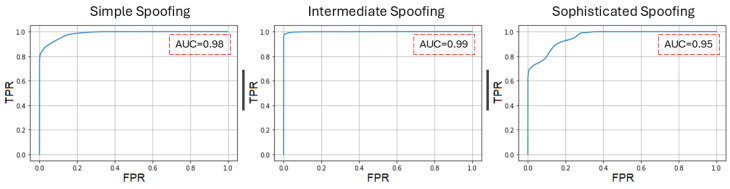
ROC curves of the AML detection model under different GPS spoofing attacks.

The performance of the proposed model was evaluated using a receiver operating characteristic (ROC) curve. Figure 11 displays the ROC curves for selected datasets with the x-axis denoting the false positive rate (FPR) and the y-axis denoting the true positive rate (TPR). The model’s detection performance was similar for datasets containing simple, intermediate, and sophisticated GPS spoofing attacks. The model’s ROC curve for simple spoofing demonstrated outstanding performance with an area under the curve (AUC) of 0.98. The model’s performance was also superior when the dataset contained intermediate spoofing with an AUC of 0.99. However, the model’s performance slightly decreased for sophisticated spoofing with an AUC of 0.95.

### 6.9. Comparative Studies

Comparative analysis of the findings leads to valuable insights into the AML detection model’s performance. Therefore, this subsection explores the consistency of the results from different studies conducted under different experimental circumstances using the same datasets.

Table 12 compares the results achieved using various approaches to GPS spoofing attacks. Aissou et al. [28] explored several tree-based ML models on a GPS spoofing attack dataset comprising 10,055 samples and employed the Spearman correlation coefficient to measure the strength of the monotonic relationship between features, enhancing feature selection. They achieved impressive results with an RF of 94.07%, GBM of 91.45%, XGB of 95.52%, and LGBM of 95.23%. Khoei et al. [36] employed a diverse set of models, including SVM, ANN, RF, GNB, CART, and LR, on a GPS spoofing attack dataset of 14,000 samples. They used Pearson’s correlation to select features and assess variable correlations. This model exhibited high accuracy across most techniques with SVM and CART achieving 98%, ANN and RF achieving 99%, and LR achieving 96%, while GNB lagged slightly at 91%. In addition, the probability of false alarms for ANN, RF, and CART demonstrated a low rate.

Our proposed AML detection model used an adversarial training method that combined GAN and CNN on a much larger set of 129,979 samples. The model used AML methodology to extract and manipulate input characteristics to achieve the desired outcome. When comparing the probability of false alarms, the proposed model in this study achieved a false alarm rate of 3%, further indicating its effectiveness and efficiency. Moreover, the proposed model achieved a detection accuracy of 98%, showcasing this innovative methodology’s effectiveness in handling large-scale datasets and complex tasks.

## 7. Limitations and Future Directions

Although the proposed model has shown encouraging results, we must acknowledge the limitations we faced. The scarcity of openly available datasets for GPS signal attacks and threats restricted the proposed model’s ability to fully comprehend the complexity of GPS signal attack behavior in authentic settings. However, the acquisition and sharing of over-the-air attack datasets are impeded by various factors, including privacy concerns, proprietary data ownership, and logistical challenges associated with data collection and organization. Overcoming these obstacles is essential to advancing research and development in wireless communication security. Addressing this difficulty requires collaborative efforts among researchers, industry stakeholders, and regulatory commissions to encourage data transparency and promote open-access datasets.

However, the findings provide opportunities for future efforts to enhance DL model-based UAV-IDSs and to overcome continuous challenges within this field. A significant scope for further investigation remains, specifically in improving the proposed model by re-evaluating its performance under different scenarios and conditions to achieve generalizability and robustness. A thorough exploration will demonstrate the proposed detection model’s reliable performance across various contexts, enhancing its efficiency and effectiveness in real-world UAV systems.

In addition, it is important to consider the necessary hardware requirements and the integration with current UAV systems to offer a more comprehensive understanding of the model’s potential deployment within existing systems. Future efforts will address the feasibility of implementing the proposed model at the UAV level.

## 8. Conclusions

This study introduces a detection model centered on AML methodology, combining the principles of physical layer security with advanced DL techniques to enhance and fortify IDS for UAVs. We propose an AML detection model to improve UAV-IDS performance by effectively classifying legitimate and malicious inputs and assessing robustness against adversarial attacks. AML training methodology seeks to improve an IDS classifier’s reliability by training it with adversarial samples. Through adversarial training, this defensive strategy fortifies the model’s resilience by incorporating a training dataset encompassing benign and adversarial samples.

Our evaluation focused on testing different DL techniques for classifying GPS signal spoofing attacks, primarily with CNN and LSTM networks. The CNN model outperformed LSTM in terms of accuracy and loss, demonstrating its effectiveness in classifying various GPS signal spoofing attacks. Integrating the AML detection model further enhanced its classification capabilities particularly when detecting sophisticated attacks. The integration resulted in an average F1 score increase of approximately 12%. Incorporating an adversarial framework into the AML detection model significantly reduced misclassified signals, improving robustness against evasion attacks and overall classification accuracy.

## Figures and Tables

**Table 1:** Information on the reviewed detection wireless signal spoofing-based machine learning approaches.

Study	Algorithms	Features	Proposed Solution	Remark
Erpek et al. [24]	GAN	Received signal strength indicator (RSSI)	Launching and mitigating wireless jamming attacks using DL	Launching jamming attack-based DL showed that this attack effectively reduces the transmitter’s throughput and success ratio compared to random and sensing-based jamming attacks.
Shi et al. [25]	GAN	Phase shift and channel I/Q	Developed a GAN-based spoofing attack	They considered both SISO and MIMO communication systems. They showed that the probability of success of this GAN-based spoofing attack is very high and holds for different network topologies and node locations.
Ma et al. [26]	LSTM	CSI	Implement an intrusion detection scheme for spoofing attacks	The performance of the proposed detection scheme is evaluated via simulations with variable channel conditions, which are conducted according to the specifications of the 5G NR architecture to verify the feasibility of the proposed solution.
Manesh et al. [27]	DNN	Satellite vehicle number, SNRP, Seudo range, Doppler shift, Carrier phase shift	Supervised machine learning detection method for GPS spoofing attacks	Different neural networks’ efficiencies with different hidden neurons were analyzed. Four metrics: probability of detection, probability of misdetection, probability of false alarm, and accuracy were used to evaluate these efficiencies.
Aissou et al. [28]	XGBoost—Light Gradient Boosting Machine—RF—Gradient Boost	13 Features	Several tree-based machine learning models to detect GPS spoofing attacks	They performed feature correlation analysis using the Spearman technique to remove correlated features and reduce the size of the dataset, resulting in a less complex and more accurate learning model.
Shafique et al. [29]	Linear regression—NB—SVM—DT—RF	Jitter, jitter (absolute), jitter (local), jitter (RAP), jitter (ppq5), shimmer, shimmer (local), shimmer (dB), shimmer (apq3), shimmer (apq5)	A security system that detects the authenticity of GPS signals	To further enhance the proposed system’s accuracy, voting techniques are integrated to choose a learning model that classifies the signal with the highest accuracy shown by a specific learning model among the other proposed learning models.
Li et al. [30]	GAN	Carrier Doppler frequency and pseudo-code phase	A GAN-based method for spoofing detection	They showed that the proposed method’s detection effect is better when the pseudo-code phase and carrier Doppler frequency of the spoofing signal are the same as those of the authentic signal.
Khoei et al. [31]	SVM, NB, DT, KNN, Linear Discriminative Analysis, RF, ANN, Logistic Regression, Elastic Net, and AdaBoost	13 features	Two dynamic-based selection methods, MOD and WMOD, to detect GPS spoofing attacks	One-stage heterogeneous ensemble feature selection was used to discard correlated and low-importance features from the considered dataset using Spearman correlation and information gain. MOD and WMOD methods dynamically select one best classifier between the implemented ML models.
Sun et al. [32]	CNN—LSTM	36 features	A method for GPS signal spoofing detection	They chose the principal component analysis method to downgrade the input GPS signal data. To address the problem of the small amount of real GPS signal data, they proposed an approach based on the SVM-SMOTE model to expand the GPS signal data.
Borhani-Darian et al. [33]	CNN	I/Q	DNN models to detect single spoofing signals	They used the cross-ambiguity function computed by GNSS receivers to detect spoofing attacks.

**Table 2:** GPS signal features description.

Feature	Description
Carrier-to-Noise Ratio (C/N°)	The ratio of the power of the carrier signal to the power of the background noise in the signal.
Prompt Correlator (PC)	The prompt correlator in a GPS receiver is designed to align the incoming received signal with the locally generated replica of the satellite’s pseudo-random code. This alignment allows the receiver to determine the time delay, or range, between the satellite and the receiver. PC can be expressed in terms of PIP and PQP components as: $PC=\sqrt{PIP^{2}+PQP^{2}}$
Early Correlator (EC)	is 1/2 chip spacing before the PC.
Late Correlator (LC)	is 1/2 chip spacing after the PC.
Prompt In-Phase Component (PIP)	The in-phase component of the prompt correlator amplitude.
Prompt Quadrature Component (PQP)	The quadrature component of the prompt correlator amplitude.
Carrier Phase Cycles (CPs)	Refers to the number of full cycles of the carrier wave that have elapsed since the GPS satellite transmitted the signal. Here, the carrier wave is a high-frequency signal generated by the GPS satellite that serves as the reference for measuring the distance between the satellite and the GPS receiver on the ground.
Time Of the Week (TOW)	It is the decoded information from the GPS signal; it provides the number of seconds elapsed since the start of each week (from 0 s to 604,799 s). It is essential in GPS because it allows receivers to determine the exact time the signal was transmitted.
Receiver Time (RX)	Refers to the time at which the GPS signal is received by a GPS receiver.
Pseudo-Range (PD)	Refers to the estimated distance between a GPS receiver and a satellite. It is not an actual geometric range but rather a measured value derived from the time it takes for the GPS signal to travel from the satellite to the receiver. It is expressed as shown in $P_{s}=c\left(t_{r}-t_{s}\right)$ where $t_{r}$ is the reception time and $t_{s}$ is the transmission time.
Carrier Doppler (DO)	The result of relative motion of the satellite with respect to the receiver known as the Doppler effect, as expressed in $f=\frac{c\;+\;v_{r}}{c\;+\;v_{s}}\times f_{i}$
Tracking Carrier Doppler (TCD)	The Doppler shift measured during the correlation stage.
Pseudo-Random Noise (PRN)	The satellite identification number.

**Table 3:** Proposed generator structure.

Layer Type	Input Size	Parameters	Activation Function
Input Shape	1,6	None	None
Dense Layer	1,6	6 neurons	linear
Reshape Layer	1,6,1	None	None
Transpose Convolutional Layer	1,12,1024	1024 neurons, kernel size 3 × 3, strides 1 × 2	linear
Convolutional Layer	1,12,1	1 neuron, kernel size 3 × 3	linear
Flatten	12	None	None
Dense Layer	13	13 neurons, kernel initializer he normal	linear

**Table 4:** Proposed discriminator structure.

Layer Type	Input Size	Parameters	Activation Function
Input Shape	1,13	None	None
Reshape Layer	1,1,13	None	None
Convolutional Layer	1,1,32	32 neurons, kernel size 3 × 3, strides 2 × 2	LeakyReLU
Convolutional Layer	1,1,16	16 neurons, kernel size 3 × 3, strides 2 × 2	LeakyReLU
Flatten	16	None	None
Dropout	16	0.4 rate	None
Dense Layer	1	1 neuron	Sigmoid

**Table 5:** Proposed CNN classifier structure.

Layer Type	Input Size	Parameters	Activation Function
Input Shape	1,13	None	None
Reshape Layer	1,1,13	None	None
Convolutional Layer	1,1,64	64 neurons, kernel size 13 × 13, strides 2 × 2	LeakyReLU
Convolutional Layer	1,1,32	32 neurons, kernel size 13 × 13, strides 2 × 2	LeakyReLU
Convolutional Layer	1,1,16	16 neurons, kernel size 13 × 13, strides 2 × 2	LeakyReLU
Flatten	16	None	None
Dropout	16	0.4 rate	None
Dense Layer	1	1 neuron	Sigmoid

**Table 6:** Proposed LSTM classifier structure.

Layer Type	Input Size	Parameters	Activation Function
Input Shape	1,13	None	None
Reshape Layer	1,1,13	None	None
LSTM Layer	1,1,64	64 neurons, return sequences = True	LeakyReLU
LSTM Layer	1,1,32	32 neurons, return sequences = True	LeakyReLU
LSTM Layer	1,1,16	16 neurons, return sequences = True	LeakyReLU
Flatten	16	None	None
Dropout	16	0.4 rate	None
Dense Layer	1	1 neuron	Sigmoid

**Table 7:** Hyperparameters used in AML detection model training.

Hyperparameter	Generator	Discriminator	CNN Classifier	LSTM Classifier
Loss Function	Mean Absolute Error	Binary Cross-Entropy	Binary Cross-Entropy	Binary Cross-Entropy
Optimizer	RMSprop	Adam	Adam	Adam
Learning Rate	0.001	0.0002	0.0002	0.0002
Batch Size	None	200	1000	1000
Dropout Rate	None	0.4	0.4	0.4
Trainable Parameters	19,638	8417	573,377	35,537

**Table 8:** IDS classifier performance corresponding to different DL techniques.

Model	K-Folds	Loss	Accuracy	Precision	Recall	F1 Score
CNN	1	0.159	0.910	0.892	0.930	0.911
	2	0.126	0.933	0.932	0.929	0.931
	3	0.114	0.949	0.962	0.932	0.947
	4	0.179	0.916	0.988	0.838	0.907
	5	0.113	0.950	0.965	0.931	0.948
LSTM	1	0.521	0.708	0.804	0.541	0.647
	2	0.534	0.707	0.791	0.541	0.642
	3	0.520	0.712	0.798	0.556	0.655
	4	0.521	0.718	0.797	0.566	0.662
	5	0.516	0.716	0.798	0.565	0.662

**Table 9:** The CNN model’s performance corresponding to different spoofed GPS signal types.

Dataset	K-Folds	Loss	Accuracy	Precision	Recall	F1 Score	TP	FP	FN	TN
Legitimate	1	0.234	0.867	0.846	0.849	0.847	6119	1117	1091	8289
+	2	0.210	0.893	0.955	0.799	0.870	5949	282	1501	8884
Simple	3	0.201	0.893	0.926	0.821	0.870	5974	477	1301	8864
Spoofing	4	0.179	0.904	0.859	0.931	0.894	6706	1100	495	8315
(LS1)	5	0.173	0.912	0.960	0.834	0.893	6110	256	1212	9037
Legitimate	1	0.147	0.920	0.975	0.861	0.915	7591	191	1225	8773
+	2	0.968	0.836	0.998	0.673	0.804	5965	9	2902	8904
Intermediate	3	0.267	0.900	0.995	0.801	0.888	7056	34	1748	8942
Spoofing	4	0.072	0.973	0.975	0.970	0.972	8585	217	270	8708
(LS2)	5	1.757	0.816	0.831	0.793	0.812	7051	1429	1839	7460
Legitimate	1	0.455	0.769	0.890	0.505	0.644	3233	399	3171	8620
+	2	0.878	0.727	1.000	0.346	0.514	2228	0	4212	8983
Sophisticated	3	0.551	0.822	0.933	0.616	0.742	3959	284	2468	8712
Spoofing	4	0.198	0.921	0.972	0.832	0.897	5270	153	1063	8937
(LS3)	5	0.136	0.946	0.969	0.898	0.932	5757	186	652	8827

**Table 10:** Performance of the proposed AML detection model corresponding to different spoofed GPS signal types.

Dataset	K-Folds	Loss	Accuracy	Precision	Recall	F1 Score	TP	FP	FN	TN
LS1 + GAN	1	0.156	0.923	0.972	0.872	0.919	8234	239	1204	9134
	2	0.136	0.937	0.943	0.931	0.937	8861	535	659	8756
	3	0.129	0.943	0.989	0.899	0.942	8581	97	967	9166
	4	0.112	0.954	0.963	0.944	0.954	8874	343	522	9072
	5	0.106	0.950	0.983	0.917	0.949	8740	150	792	9128
LS2 + GAN	1	0.107	0.951	0.935	0.978	0.956	10,408	720	235	8175
	2	0.071	0.972	0.965	0.984	0.974	10,464	383	170	8521
	3	0.077	0.968	0.960	0.983	0.971	10,385	432	184	8537
	4	0.043	0.979	0.997	0.964	0.980	10,130	30	373	9005
	5	0.050	0.977	0.991	0.966	0.978	10,310	90	364	8774
LS3 + GAN	1	0.092	0.965	0.994	0.933	0.963	7745	46	553	8914
	2	0.095	0.962	0.979	0.940	0.959	7727	164	496	8871
	3	0.092	0.958	0.948	0.965	0.956	7946	439	285	8588
	4	0.376	0.917	0.883	0.951	0.916	7803	1029	398	8028
	5	0.097	0.954	0.992	0.911	0.950	7506	58	729	8964

**Table 11:** Evaluation of the detection model following implementation of the offensive model.

Applying AML Model	Time	Loss	Accuracy	Precision	Recall	TP	FP	FN	TN
Before	0 s 1 ms/step	2.8437	0.626	1	0.626	1252	0	748	0
After	0 s 3 ms/step	0.0442	0.984	1	0.984	1968	0	32	0

**Table 12:** Comparison between studies conducted on the GPS spoofing attack dataset and the proposed AML detection model.

Approach	Model Techniques	Dataset Size	Feature Selection Techniques	Accuracy	Probabilities of False Alarm
Aissou et al. [28]	RF–Gradient Boost (GBM)–XGBoost (XGB)–Light Gradient Boosting (LGBM)	10,055 samples	To express how strong the monotonic relation is between the features, they used the Spearman correlation coefficient.	RF = 94.07% GBM = 91.45% XGB = 95.52% LGBM = 95.23%	RF = 9% GBM = 10% XGB = 4% LGBM = 5%
Khoei et al. [36]	SVM-ANN–RF–Gaussian Naïve Bayes (GNB)–Regression Decision Tree (CART)–Logistic Regression (LR)	14,000 samples	To predict how well the variables are correlated, they used Pearson’s correlation.	SVM = 96% ANN = 98% RF = 98% GNB = 91% CART = 99% LR = 91%	SVM = 7% ANN = 3% RF = 2% GNB = 4% CART = 2% LR = 5%
AML Detection Model	GAN + CNN	129,979 samples	AML methodology to extract information about the behavior and characteristics of inputs and how to manipulate them to obtain a desired outcome.	The detection accuracy is 98%	3%

## Data Availability

The data presented in this study are available on request from the corresponding author.

## Abbreviations

The following abbreviations are used in this manuscript:

UAV	Unmanned aerial vehicles
GPS	Global Positioning System
AML	Adversarial Machine Learning
DL	Deep learning
DNN	Deep neural network
GAN	Generative adversarial network
CNN	Convolutional neural network
LSTM	Long short-term memory
